# DFTSA-Net: Deep Feature Transfer-Based Stacked Autoencoder Network for DME Diagnosis

**DOI:** 10.3390/e23101251

**Published:** 2021-09-26

**Authors:** Ghada Atteia, Nagwan Abdel Samee, Hassan Zohair Hassan

**Affiliations:** 1 Information Technology Department, College of Computer and Information Sciences, Princess Nourah Bint Abdulrahman University, Riyadh 11461, Saudi Arabia; nmabdelsamee@pnu.edu.sa; 2 Computer Engineering Department, Misr University for Science and Technology, Giza 12511, Egypt; 3 Department of Mechanical Engineering, College of Engineering, Alfaisal University, Takhassusi Street, P.O. Box 50927, Riyadh 11533, Saudi Arabia; hzahmed@alfaisal.edu

**Keywords:** diabetic macular edema, retinal fundus image, deep learning, pretrained convolutional neural network, autoencoder, transfer learning

## Abstract

Diabetic macular edema (DME) is the most common cause of irreversible vision loss in diabetes patients. Early diagnosis of DME is necessary for effective treatment of the disease. Visual detection of DME in retinal screening images by ophthalmologists is a time-consuming process. Recently, many computer-aided diagnosis systems have been developed to assist doctors by detecting DME automatically. In this paper, a new deep feature transfer-based stacked autoencoder neural network system is proposed for the automatic diagnosis of DME in fundus images. The proposed system integrates the power of pretrained convolutional neural networks as automatic feature extractors with the power of stacked autoencoders in feature selection and classification. Moreover, the system enables extracting a large set of features from a small input dataset using four standard pretrained deep networks: ResNet-50, SqueezeNet, Inception-v3, and GoogLeNet. The most informative features are then selected by a stacked autoencoder neural network. The stacked network is trained in a semi-supervised manner and is used for the classification of DME. It is found that the introduced system achieves a maximum classification accuracy of 96.8%, sensitivity of 97.5%, and specificity of 95.5%. The proposed system shows a superior performance over the original pretrained network classifiers and state-of-the-art findings.

## 1. Introduction

Diabetes is one of the chronic diseases which, if not controlled, causes damage to the eyes, kidneys, blood vessels, and body nerves. According to the World Health Organization (WHO), nearly 422 million people worldwide have diabetes, and nearly 1.6 million people die every year as a result of the complications caused by this disease [1]. Due to diabetes, the retina in diabetic patients is affected by a microvascular complication which is called diabetic retinopathy (DR). Prolonged poor control of blood glucose levels could eventually cause damage to the small blood vessels in the eye. This damage ultimately results in the leakage of fluids into the retina [2]. As the DR continues, the accumulation of extracellular fluids in the macula causes it to swell. The condition when the macula swells with fluids in a DR patient is called diabetic macular edema [3]. Hard exudates (HEs) and hemorrhages are DME-associated lesions that have been used to identify the existence of the disease. Depending on whether the distance between the HEs and the center of the macula is less or greater than an optic disc diameter, the disease is categorized as mild or severe DME, respectively [4].

The macula is responsible for central vision in the eye. Therefore, when the macula is affected with edema, vision starts to blur and could be entirely lost. It is noteworthy that, according to the WHO [5], about 3.9 million people in the world suffer from vision impairment or blindness that is caused by diabetic retinopathy. DME is considered the most common cause of permanent visual impairment in diabetes patients with DR if it is not diagnosed and treated [6]. Unfortunately, the early stages of DME usually develop without observable symptoms, especially when the edema is not centered in the macula [6,7]. As a consequence, the patient is often unaware of having DME disease. As the edema extends to the central macula, vision starts to deteriorate progressively in a relatively short span of time [7]. Therefore, early diagnosis of DME is pivotal for the timely treatment of the disease. To avoid the aforementioned complications, ophthalmologists recommend that diabetes patients perform eye examinations at regular intervals of time.

Diabetic macular edema can be diagnosed through clinical examinations as well as eye screening. In clinical eye examination, the pupil is dilated using specialized eye drops, and the retina is investigated manually by the opthalmologist. As the diabetic population increases, more eye examinations are required to be checked by ophthalmologists to diagnose diabetes-related eye diseases including DME. Manual evaluation of DME through clinical examination is a time-consuming process and may lead to delayed diagnosis and treatment of this critical disease. Recently, eye screening techniques such as fluorescein angiography, fundus photography, and optical coherence tomography (OCT) have been considered effective tools that could assist specialists in diagnosing the presence of DME as well as its progress. To reduce the burden on eye specialists in manually investigating patients’ screening images, automated image-based diagnosis techniques are urgently required. This way, specialists’ time and effort will be devoted to treating more advanced cases, and to containing the growth of DME disease. Furthermore, including automatic DME diagnosis systems in the triaging systems of eye clinics could effectively enhance the healthcare services.

The recent evolution of machine learning technologies, particularly deep learning, has facilitated the automation of services in several life aspects. The use of machine and deep learning techniques has shown significant prominence in the medical field. For instance, machine learning has been used effectively for monitoring the vital signs of patients to develop a personal profile based on these signs [8]. Deep learning techniques have been utilized to predict people with diabetes based on patients’ information such as the blood glucose level, blood pressure, and others [9]. Moreover, deep convolutional neural networks (CNNs) have been considered the base to develop many computer-aided diagnosis (CAD) systems. Recently, CAD systems have been utilized to automatically identify the existence of many diseases such as DR, DME, different types of tumors, and COVID-19. Previous studies have shown variation in the performance of the available CAD systems. This variation in performance is dependent on the type and design of the underlying deep learning algorithm as well as other factors such as the image quality.

In a contribution to provide a competing automatic DME diagnosis system with high performance, a novel deep feature transfer-based DME classification system for retinal fundus images is proposed in the present work. The introduced system is based on the integration of two powerful deep learning tools, namely, pretrained CNNs and autoencoder networks. Standard pretrained CNNs are utilized to extract features from the input images. These features are combined together to build an enlarged feature set to promote a better classification performance. The autoencoders are used to develop a stacked network to select the most significant features and perform DME classification. The framework of the proposed system consists of four phases: data preprocessing, feature extraction and integration, feature selection, and DME classification. The major contributions of the current study are summarized as follows:

1. Development of a new system, DFTSA-Net, based on integrating pretrained CNNs with autoencoder networks for the automatic diagnosis of DME in fundus images;
2. Preprocessing of input images for quality enhancement;
3. Utilization of a number of off-the-shelf pretrained CNNs to extract deep features from the input images;
4. Creation of an enlarged feature dataset from a limited size dataset to improve the classification performance;
5. Selection of the most significant features from the enlarged feature set using autoencoder networks through unsupervised learning;
6. Development and training of stacked autoencoder network in a semi-supervised manner to diagnose DME;
7. Investigation of the effect of input feature set size on the performance of the proposed DFTSA-Net system.

The rest of this paper is organized as follows: Section 2 presents a literature review related to the existing techniques of diagnosing DME, Section 3 introduces the used data and the proposed methodology, Section 4 describes the experimental results and discussion, and Section 5 concludes the work.

## 2. Literature Review

Automatic diagnosis of DME in retinal photographs has become an urgent demand in light of the recent increasing pervasiveness of diabetes. In response to this demand, many research groups have tackled the problem of automatic detection of DME in retinal screening images from several aspects. Methods used for diagnosing DME in previous studies could be classified into two groups: image processing-based methods, and classification-based methods. Image processing-based methods search for exudates in the image to diagnose DME [10,11]. These methods utilize image enhancement and noise removal techniques to enhance the quality of retinal images [10]. The enhanced image is primarily segmented to identify retinal objects such as the optic disc, the macula, and the blood vessels. DME-related lesions such as exudates are segmented afterwards. Common segmentation algorithms usually combine noise filters (such as the Gaussian, median, and other filters), edge detection algorithms (such as Canny or Sobel detectors), local or global thresholding, and morphological operations. The presence or absence of DME is declared based on the lesion segmentation results. For instance, Sánchez et al. [12] developed a dynamic thresholding technique based on a mixture model estimated from the histogram of an enhanced green component of an RGB retinal image to separate exudates. Although they obtained a sensitivity of 90.2%, some bright marks such as optical artifacts and blood vessels were misclassified as exudates. Walter et al. [13] developed a framework to detect exudates based on mathematical morphology. They employed watershed transformation and morphological filtering to locate and exclude the optic disc. They used the variation in the gray level intensity to locate exudates. An algorithm was developed by Sopharak et al. [14] for segmenting exudates from low-contrast retinal images based on fuzzy C-means (FCM) clustering and morphological operation. In that work, a number of features were extracted from the input images and fed to a coarse segmentation step using FCM clustering. Sobel edge detection, morphological operations, and thresholding were used to extract the required features. They obtained a sensitivity and specificity of 87.2% and 99.2%, respectively.

Classification-based methods utilize machine learning techniques to recognize normal images from DME-diseased images. Classification algorithms use image-based features (such as texture, shape, or image statistics) to assign a class to the input image. In the case of a poor image quality, it would be important to preprocess images before extracting features. The process of feature extraction could be achieved manually by calculating features such as the region of interest area, perimeter, mean, and variance of the pixel intensity, among others [10]. Features can also be extracted automatically using deep learning networks [15,16] which accelerate the time-consuming process of feature handcrafting.

According to the type of used machine learning classification algorithm, classification-based methods can be divided into conventional classification methods and deep learning methods. Conventional classifiers (such as support vector machines (SVMs), random forest, and k-nearest neighbors) are basically fed with hand-crafted features. Such classifiers could perform reasonably well with a limited dataset size at a low computational cost. However, the performance of conventional classifiers is highly impacted by the selection of hand-crafted features. On the other hand, deep neural networks require larger training datasets, extract features automatically, and generally provide a higher classification performance at a higher computational cost [15,16,17,18,19,20]. The classical SVM classifier was used by Shengchun et al. [21] for automatic hard exudate classification. They employed a dynamic threshold and fuzzy C-means clustering for finding candidates of hard exudates. The features were extracted from the hard exudate candidates and fed to an SVM classifier. Using that algorithm, the authors obtained an F1-score of 76.7% on the e-ophtha EX database and an accuracy of 97.7% on the DIARETDB1 database. In another work [22], an automatic DME classification framework was developed based on extracting the histogram of oriented gradients and local binary pattern features from spectral domain OCT images. Features were selected by principal component analysis (PCA), and the classification was performed using a linear support vector machine. This approach recorded a sensitivity and a specificity of 87.5%.

Recently, many studies have undertaken the problem of DME diagnosis using deep neural networks. For instance, Al-Bander et al. [16] developed an advanced CNN that extracted features to detect the severity of DME disease using the MESSIDOR dataset. Their results showed an accuracy of 88.8%, sensitivity of 74.7%, and specificity of 96.5%. Singh et al. [23] developed a hierarchical ensemble CNN (HE-CNN) model to detect DME. They adopted a preprocessing step on color fundus images using a morphological opening and Gaussian kernel. For the IDRiD and MESSIDOR datasets, their work presented an accuracy of 96.12%, sensitivity of 96.32%, and specificity of 95.84%. The work conducted by Mo et al. [24] proposed a DME system that is composed of two cascaded deep residual networks to recognize DME. In that study, the first fully convolutional residual network fused multi-level hierarchical information to segment exudates accurately from the input images. Based on the segmentation results, the region centered on the pixel with the highest likelihood was cropped and fed into the second deep residual network which was used for DME classification. This model achieved a sensitivity of 96.3%, specificity of 93.04%, and accuracy of 94.08% on the HEI-MED dataset. Sulaiman et al. [25] developed a deep learning-based DME-grading model which consists of an autoencoder network and a DME-grading network. The autoencoder network was pretrained using the Kaggle dataset of fundus images and was used to learn features of input images. The DME-grading network detected and graded the risk of DME. The highest obtained results using the IDRiD were 68% for the accuracy, 66% for the precision, 68% for the sensitivity, and 65% for the F1-score. Another method based on a convolutional neural network with an encoder–decoder architecture was developed to automatically detect and quantify intraretinal cystoid fluid (IRC) and subretinal fluid in OCT images [26]. The encoder maps an OCT image to an abstract representation and then the decoder maps this representation to a full-input resolution label image. After that, a quantification of the detected fluid to age-related macular degeneration, diabetic macular edema, or retinal vein occlusion is conducted. They found that their method achieved optimal accuracy for the detection and quantification of IRC for all three macular pathologies, with a mean accuracy of 94% [26]. Although deep networks offer a satisfactory classification performance, their processing is computationally extensive and time-consuming. Therefore, a number of recent studies started using deep learning networks for automatic feature extraction instead of utilizing deep networks for classification in several disciplines including the medical field [27].

One of the recent classes of deep networks is the pretrained image classification convolutional neural network class. This class was trained by an extremely large dataset of natural images, namely, the ImageNet database [28]. Pretrained CNNs can be used as automatic feature extractors or as image classifiers through transfer learning. In transfer learning, the final layers of a pretrained network are fine-tuned to perform a specified classification problem on a new dataset. Karri et al. [29] used this technique for identifying DME in OCT images and showed that pretrained CNN models can be fine-tuned for classifying non-medical images with limited training data. They fine-tuned the GoogLeNet CNN [30] model to diagnose DME and obtained higher accuracy compared to traditional classification methods. A large-scale study was conducted by Tang et al. [31], where two versions of a multitask ResNet-34 CNN were presented to diagnose DME. They used three-dimensional and two-dimensional OCT scans, with a total of 73,746 and 26,981 scans for training and evaluating the deep network, respectively. This system achieved a high classification performance with area under the receiver operating characteristic curves of 0.94, 0.96, and 0.97 for the CIRRUS, SPECTRALIS, and Triton OCT datasets, respectively. Although promising results have been obtained from using transfer learning for classifying images, the classification performance is still comparable to classical classifiers [17].

Aiming to further improve the classification performance, a few recent papers employed some common off-the-shelf pretrained CNNs to automatically extract features from input images and then fed them to classical classifiers to recognize diabetic eye diseases. Mohamed et al. [32] used the feature map of the ResNet-50 network [33] as an input to a random forest classifier for DR diagnosis in fundus images. Their system achieved an accuracy of 96% and 75.09% on the MESSIDOR-2 and EyePACS datasets, respectively. They found that their system provided higher accuracy than that obtained using the fine-tuned ResNet-50 network and other pretrained CNNs. In another work [34], a framework based on deep learning was created for DME recognition on OCT images through transfer learning. The authors first denoised and cropped the input images, and then features were extracted using AlexNet [28] and passed to an SVM classifier. Their experiments showed that denoising and cropping images lead to a better classification accuracy of 96%. Muhammad et al. [19] used the pretrained VGG-16 CNN for extracting features of OCT images at different layers of the network and then passed these features to different classical classifiers for DME classification. Their results presented an accuracy of 87.5%, with a sensitivity of 93.5%, and specificity of 81%. Chan et al. [35] discussed the issue of transferred feature reduction for DME classification in OCT images. In that work, an AlexNet-based model for the classification of DME was used to extract features from OCT images. The PCA and bag-of-words methods were used for feature reduction, and linear SVM was used for classification. Their results revealed that the PCA associated with deep features showed very good performance, with an accuracy of 96.8% and sensitivity of 93.75%; however, the bag-of-words showed poor performance, with an accuracy of 81.25%. Abbas [17] developed a modified dense convolutional neural network (DCNN) model to diagnose DME disease. The DCNN model was developed by adding five convolutional layers and one dropout layer to the original pretrained Dense CNN network. The author trained and tested the DCNN model using the Hamilton HEI-MED, IDRiD, and MESSIDOR datasets. The DCNN model achieved an accuracy of 91.2%, specificity of 94.4%, and sensitivity of 87.5%. A custom deep learning-based CenterNet model was developed by Nazir et al. [36] for various DR lesions’ classification. Their method involved dataset preparation and feature extraction using DenseNet-100. Annotations for suspected lesions were generated. The annotated images were used to train the customized CenterNet model to localize and classify the lesions. Their model recorded an average accuracy of 97.93% and 98.10% for the APTOS-2019 and IDRiD datasets, respectively.

The aforementioned studies and others have provided encouraging results, although the effectiveness of using feature transfer learning for DME classification has not yet been comprehensively discussed in the literature. In other words, the usefulness of feature transfer learning has been widely studied when used with classical classifiers. However, the effectiveness of using feature transfer learning through the common ready-to-use pretrained CNNs with neural network classifiers for the diagnosis of diabetic maculopathy requires more investigation. Furthermore, it can be noticed that most studies were conducted on OCT images, and there is a lack of such studies on fundus images. Color fundus photography of the retina shows signs of DME and is considered the most affordable type of eye screening for the majority of patients, especially in poor communities. Inspired by these ideas, a new framework that integrates pretrained CNNs with autoencoder networks to develop a deep feature transfer-based classification system is introduced for the diagnosis of DME in fundus images.

## 3. Materials and Methods

In this section, a short description of the used dataset is presented first, and the proposed system is explained afterwards.

### 3.1. Data

**Figure 1:**
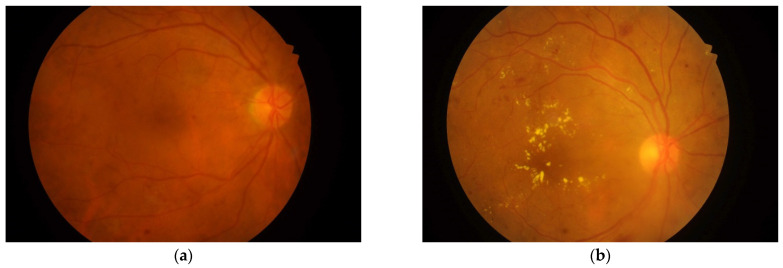
Fundus images of retinas from the IDRiD dataset [37]: (**a**) normal (No DME); (**b**) with DME signs.

The data used in this research are fundus retinal images from the Indian Diabetic Retinopathy Image Dataset (IDRiD) [37]. The dataset consists of 516 images categorized as images with DME signs, or normal images (with no signs of DME), as shown in Figure 1. A Kowa VX-10α digital fundus camera with a 50° field of view was used to capture the images. The image resolution is 4288 × 2848 pixels. The dataset provides ground truths associated with the signs of DME and the normal retinal structure. The data also provide the macular edema severity level as 0 for No DME, 1 for Mild DME, and 2 for Severe DME. In this work, we considered that the images with grading levels 1 and 2 belong to a single DME (positive) class, and images with level 0 belong to a Non-DME (negative) class. After this rearrangement, we obtained a total 294 DME images and 222 normal images.

### 3.2. Methods

**Figure 2:**
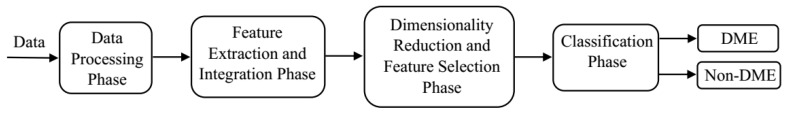
The proposed DFTSA-Net DME diagnosis system.

The proposed system consists of four phases, as depicted in Figure 2: data preprocessing, feature extraction and integration, dimensionality reduction and feature selection, classification (DME/Non-DME). First, a short description of the used data is presented, and then each of the phases is explained afterwards.

#### 3.2.1. Data Preprocessing

**Figure 3:**
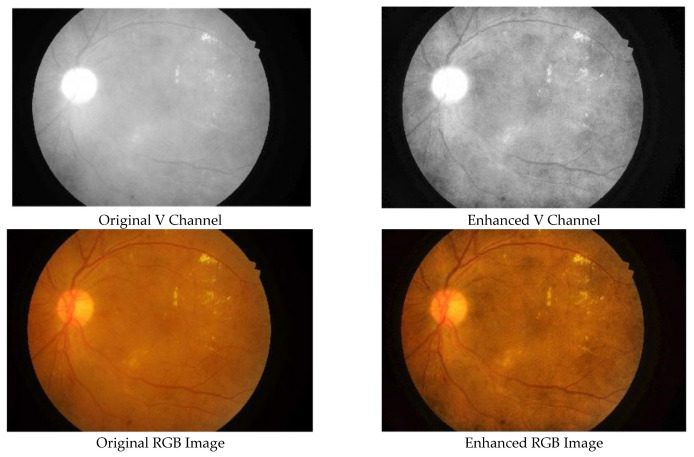
Original fundus image versus enhanced image: (**upper row**) V channel of HSV images; (**lower row**) RGB images.

Initially, the input RGB image is converted to an HSV image, and then the contrast-limited adaptive histogram equalization (CLAHE) technique is used to enhance the contrast between the background and foreground in the V channel. The parameters of the CLAHE are set to a total number of tiles of 64 and a clip limit of 0.005. These values achieve the required contrast enhancement and were determined through experimentation. The original S and H channels are kept unprocessed and then are combined with the enhanced V channel to form the enhanced HSV image, which is then converted to the RGB domain. Figure 3 illustrates the original versus enhanced images.

**Figure 4:**
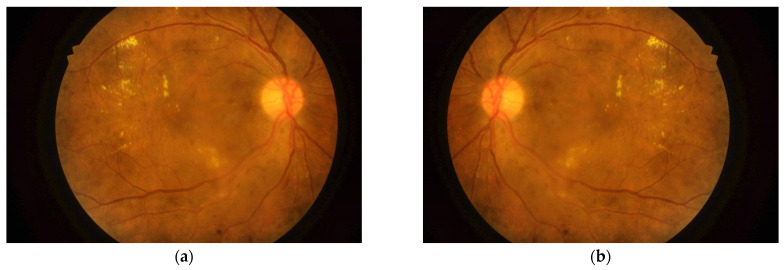
Horizontal reflection of retinal images for data augmentation: (**a**) original image; (**b**) horizontally reflected image.

Transfer learning classification of DME using pretrained CNNs was used as a reference for evaluating the performance of the proposed DFTSA-Net system. Nevertheless, the number of available input images is considered limited to provide a good classification performance from the deep learning networks. For this reason, and to avoid overfitting as well, the input dataset was augmented in the present work. Data augmentation expands the image dataset by adding more images that have variations to the original images and including them in the input dataset. Several image transformations could be used for data augmentation such as shifting, scaling, cropping, rotation, and reflections. In the present work, horizontal reflection was adopted to augment the data. In horizontal reflection, all image columns are flipped around the vertical axis, as shown in Figure 4. Training a deep learning model with an augmented dataset has been shown to improve the performance of the model, reduce overfitting, and increase the model’s ability to generalize [36].

**Figure 5:**
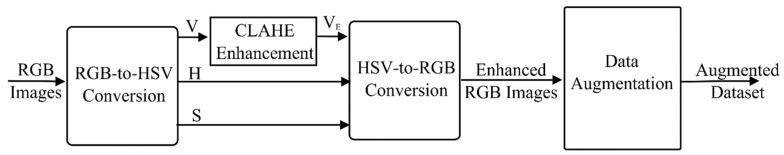
Data preprocessing phase.

In order to balance the augmented dataset, the same number of images for each class was used, providing a total number of 888 images. Moreover, six-fold cross validation was performed, and thus the five training folds combined form 740 images, while the testing fold contains 148 images. The training and testing sets represent 83% and 17% of the input dataset, respectively. The data preprocessing phase is depicted in Figure 5.

#### 3.2.2. Feature Extraction and Integration

**Figure 6:**
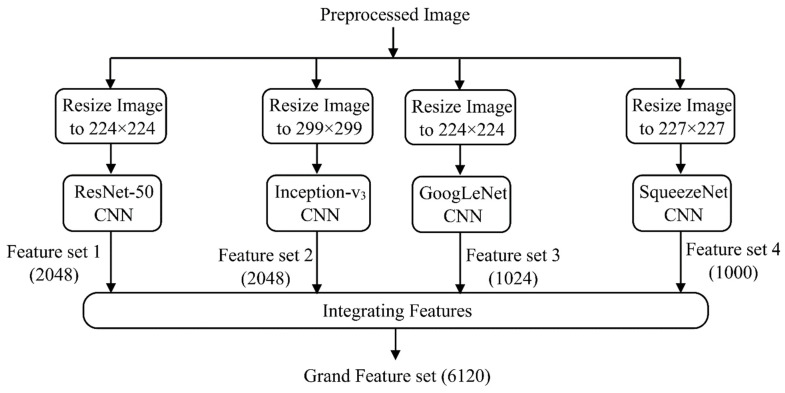
Feature extraction and integration phase.

In this phase, learned image features are extracted using a number of common pretrained CNNs, and then those features are combined to train a stacked autoencoder classifier. With this strategy, an increased number of deep features are extracted automatically from a relatively small dataset. The input dataset is passed to GoogLeNet, Inception-v3, ResNet-50, and SqueezeNet to extract deep features. These pretrained CNNs and others were trained using a subgroup of the ImageNet dataset [28] and have learned to extract useful features from the images of the dataset. Moreover, the process of feature extraction using these networks consumes less time than training the deep network for classification. The characteristics of the used networks are depicted in Table 1. The image size is modified to the designated size of the network’s input image before the images are fed to the network, as shown in Figure 6. The activations of the global pooling layer near the end of each network are then used to extract the deep features. The global pooling layer groups the input features over all the network spatial locations. The total number of features per image for each network is depicted in Table 1. Each network provides one feature set for the input dataset. Afterwards, the four feature sets are combined into a single grand feature set (GFS) for the entire dataset. The GFS is then split into the training and testing sets. We denote the GFS of the training set as (GFS)_Train_ which is [740 × 6120] in size, while that for the testing set as (GFS)_Test_, with the size [148 × 6120]. The GFS is passed to the next phase for dimensionality reduction.

#### 3.2.3. Dimensionality Reduction and Feature Selection

In this phase, a representative subset of the deep features is selected to be used for DME classification. Herein, two cascaded autoencoder neural networks are used for the task of dimensionality reduction. Composed of an encoder–decoder structure, the autoencoder is a neural network that is basically used to learn a compressed representation of the input data [38]. The encoder maps the input to a compressed representation and the decoder attempts to reverse the mapping to regenerate the input. To use the autoencoder as a dimensionality reduction tool, the autoencoder is fed with an input (the GFS in this case), and the number of neurons in the hidden layer is set to be less than the size of the input. In the present work, sparse autoencoders were trained in an unsupervised fashion (i.e., without using the training labels). Sparsity was incorporated in the autoencoders by adding a regularizer for the neurons’ activations to the cost function, as given by the following equation [39]:

(1) $$E=\underset{\underset{mean\;squared\;error}{⏟}}{\underset{\;}{\frac{1}{N}\;\overset{N}{\underset{n=1}{\sum }}\overset{K}{\underset{k=1}{\sum }}{\left(x_{kn}-{\hat{x}}_{kn}\right)}^{2}}}+\lambda \times \Omega_{weights}+\beta \times \Omega_{sparsity}$$

where *N* is the number of observations in the training data, *K* is the number of variables in the training data, x is a training example, and $\hat{x}$ is the estimate of the training example. As shown in Equation (1), the autoencoder cost function E is defined as the mean squared error function adjusted to include two terms: the sparsity regularization, $\Omega_{sparsity},$ and the weight regularization, $\Omega_{weights}$ [39]. The sparsity regularizer constrains the output value from a neuron to be low, enabling the autoencoder to learn a representation from a small subset of the training examples. The impact of the sparsity regularizer on the cost function depends on the value of its coefficient, denoted as *β* in Equation (1). The weight regularization term prevents the values of the neuron weights from increasing which consequently could decrease the sparsity regularizer. The coefficient of the weight regularizer is denoted as *λ* in Equation (1).

The mathematical representations of $\Omega_{sparsity}$ and $\Omega_{weights}$ are given by the following expressions:

(2) $$\Omega_{weights}=\;\frac{1}{N}\;\overset{L}{\underset{l}{\sum }}\overset{N}{\underset{j}{\sum }}\overset{K}{\underset{i}{\sum }}{\left(w_{ji}^{\left(l\right)}\right)}^{2}$$

where L is the number of hidden layers, and w is the weight of the corresponding neuron according to the counters i,j,l [39].

(3) $$\Omega_{sparsity}=\overset{D^{\left(1\right)}}{\underset{i=1}{\sum }}KL\;\left(\;\rho ||{\hat{\rho }}_{i}\right)\;=\overset{D^{\left(1\right)}}{\underset{i=1}{\sum }}\rho \;log\left(\;\frac{\rho }{{\hat{\rho }}_{i}}\right)\;+\left(1-\rho \right)\;log\left(\;\frac{1-\;\rho }{1-{\hat{\rho }}_{i}}\right)\;\;\;\;$$

where ${\hat{\rho }}_{i}$ is the average activation of a neuron i, ρ is the desired average activation of the neurons in the first layer ($D^{\left(1\right)})$, and $KL\;\left(\;\rho ||{\hat{\rho }}_{i}\right)$ is the Kullback–Leibler divergence between *ρ* and ${\hat{\rho }}_{i}$ [39].

The autoencoders are trained using the scaled conjugate gradient algorithm (SCGA) [40]. The training process stops when either the gradient reaches a minimum of $1\times 10^{-6}$ or the epochs reach a maximum number of 1000.

**Figure 7:**
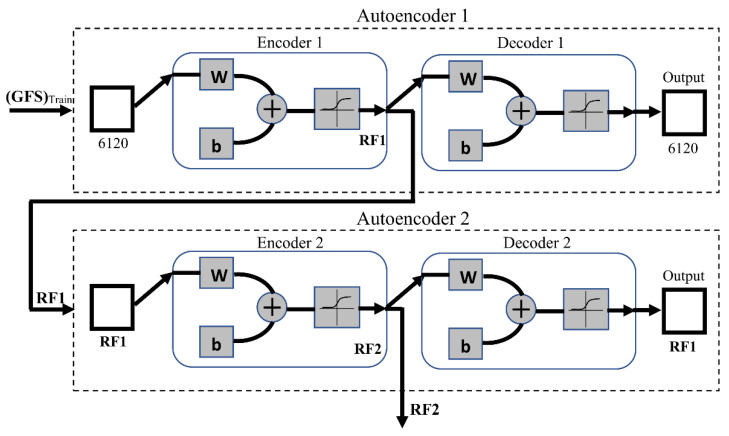
Cascaded autoencoders for feature dimensionality reduction. ‘W’ is the weight matrix and ‘b’ is the bias vector of the neurons in a network layer.

In order to execute this phase, the first autoencoder (Autoencoder 1) is trained on the (GFS)_Train_ as the input training set. In this work, the sparsity proportion, which determines the desired average activation of the encoder layer ρ, was set equal to 0.01, and the other parameters were as follows: N=740, K=6120, λ=0.004, β=4. The transfer function used for the first encoder (Encoder 1) is the positive saturating linear transfer given as in Equation (2), while the ordinary linear transfer function is used for the first decoder (Decoder 1). After training Autoencoder 1 using the entire training set, the feature set is extracted from Encoder 1, and Decoder 1 is discarded. Throughout the paper, this set will be referred to as Reduced Feature Set 1 (RF1). The second autoencoder (Autoencoder 2) is trained in the same way, but RF1 is used as the training input. To obtain a smaller feature set at the output, the size of the hidden representation of Autoencoder 2 is decreased to be half the size of RF1. For this experiment, the sparsity proportion was set equal to 0.1, and the other parameters were as follows: N=740, K=RF1, λ=0.002, β=4. The logistic sigmoid function as in Equation (3) is used as the transfer function for both the second encoder (Encoder 2) and decoder (Decoder 2). After training, a second reduced set of features from Encoder 2 is extracted and Decoder 2 is discarded. This set is called Reduced Feature Set 2 (RF2). Many experiments were conducted to set the values of all the above parameters, and the recorded ones were considered as they achieve the best classification performance. This phase is demonstrated in Figure 7.

(4) $$f\left(z\right)=\{\begin{array}{ll} 0, & if\;z\;\leq 0\\ z, & if\;0<z<1\\ 1, & if\;z\geq 1 \end{array}$$

(5) $$f\left(z\right)=\frac{1}{1+e^{-z}}$$

where z is the input to the function f.

#### 3.2.4. DME Classification

**Figure 8:**
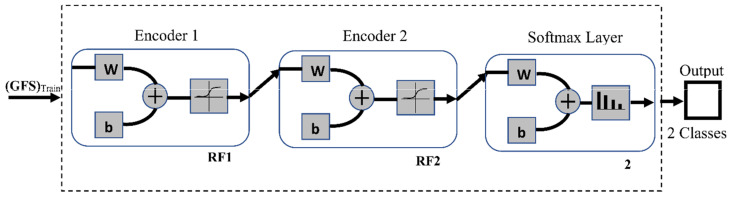
Stacked autoencoder neural network DME classifier. ‘W’ is the weight matrix and ‘b’ is the bias vector of the neurons in a network layer.

**Figure 9:**
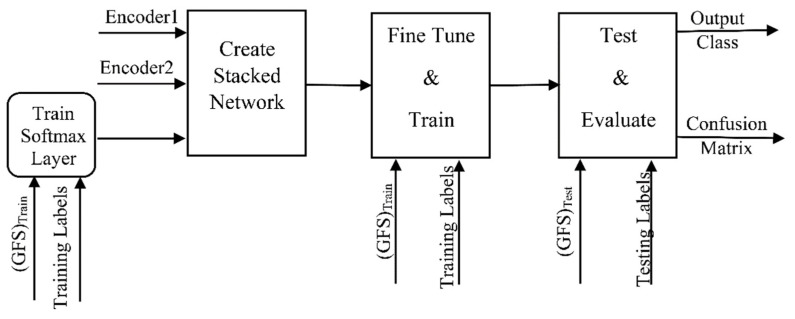
Classification phase using a stacked autoencoder neural network.

To construct the DME classifier, the previously trained encoders from Autoencoders 1 and 2 are joined to a softmax layer to form the stacked network classifier shown in Figure 8. Unlike the encoders, the softmax layer is trained in a supervised manner using the RF2 set and its training labels. The stacked network is then fine-tuned by retraining the whole multilayer network in a supervised fashion using the training GFS and its labels. The stochastic gradient descent with momentum (SGM) algorithm is used for the training. The training process stops when either the gradient reaches a minimum of $1\times 10^{-6}$ or the epochs reach a maximum number of 1000. The classification phase is illustrated in Figure 9.

#### 3.2.5. Classification Performance Evaluation

Finally, the system is tested using the testing data, and its performance is evaluated with 6-fold cross validation. The confusion matrix is generated and used to calculate a number of performance metrics. As a medical application, it is critical that the classifier is able to correctly identify the presence or absence of the disease. Therefore, the sensitivity (SN) and the specificity (SP) are considered the most important performance measures and were used in this study. While the sensitivity reflects the ability of the classifier to correctly identify images with the disease, the specificity reflects the ability of the classifier to correctly identify images without the disease [41].

Classification accuracy (AC) is defined as the ratio of images that are classified correctly [41]. A large class imbalance can highly impact the reliability of this metric for evaluating classification models. Nevertheless, in this work, as the number of images for the normal and abnormal classes is equal in the used dataset, the accuracy does reflect the real performance of the classifier. Therefore, the AC is reported in the results of the present study as well. Sensitivity, specificity, and accuracy are computed as in Equations (6)–(8), respectively [41].

(6) $$SN=\frac{TP}{TP+FN}$$

(7) $$SP=\frac{TN}{TN+FP}$$

(8) $$AC=\frac{TP+TN}{TN+TP+FN+FP}$$

where TN is the number of true negatives, TP is the number of true positives, FN is the number of false negatives, and FP is the number of false positives.

## 4. Results and Discussion

Based on the introduced methodology, the training data were preprocessed and passed through the GoogLeNet, ResNet-50, Inception-v3, and SqueezeNet CNNs individually for deep feature extraction. The four feature sets were then combined together forming the GFS. The training GFS was used to train Autoencoder 1 in an unsupervised fashion, and the first reduced feature set RF1 was used for the unsupervised training of Autoencoder 2. The second reduced feature set RF2 and its training labels were used to train a softmax layer. The entire stacked autoencoder network which consists of Encoder 1, Encoder 2, and the softmax layer was retrained in a supervised manner to be fine-tuned to the training dataset through backpropagation. The system was tested using the testing dataset, and the confusion matrix was used to compute the specificity, sensitivity, and accuracy classification measures. Based on the introduced methods, a computer code was constructed in Matlab to implement the presented DME diagnosis system. Experiments were tested on an Intel^®^ Core i7-8550U CPU with 16 GB of RAM and running Windows 10 Pro 64-bit.

**Figure 10:**
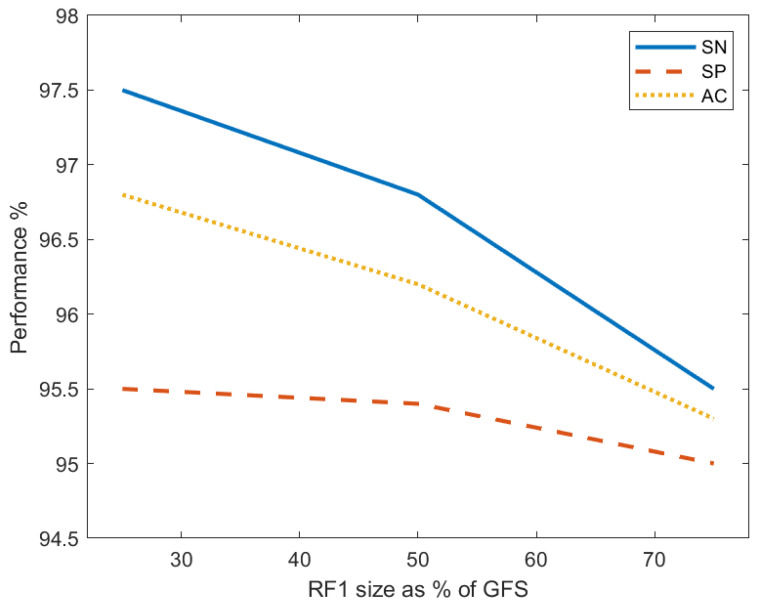
The relation between the percent sensitivity (SN), specificity (SP), and accuracy (AC) and the size of the first reduced feature set (RF1) as a percentage of the size of the grand feature set (GFS).

Three experiments were conducted to investigate the effect of the first reduced feature set (RF1) size on the system classification performance. Three models were generated, each of which has a different RF1 size and a different number of features in RF1 and RF2. Autoencoder 1 was always trained with the entire training GFS which contains 6210 feature per image. The size of RF1 was set to 75%, 50%, and 25% of the GFS size for the first model (M1), the second model (M2), and the third model (M3), respectively. In all experiments, the RF2 size was set as 50% of the RF1 size, which was used to train Autoencoder 2. The entire stacked network was then built and retrained using these feature sets and their labels in each experiment. Table 2 records the performance metrics for the proposed system of the three models and demonstrates the corresponding number of features of RF1 and RF2. Model M1 achieves a sensitivity of 97.5%, specificity of 95.5%, and accuracy of 96.8%. These obtained values reflect the highest performance over all other models. To study the behavior of the performance metrics in response to changing the size of RF1, the sensitivity, specificity, and accuracy are plotted against the RF1 size for the three models in Figure 10. The plots depict that SN, SP, and AC all decrease with the drop in the RF1 size. It can be noticed that the specificity slowly decreases as the RF1 size decreases; however, the sensitivity and accuracy drop more steeply. This decreasing behavior of the performance measures proves the effectiveness of enlarging the feature set of the input images in improving the classification performance.

To evaluate the classification performance of the proposed system against that of the original pretrained networks, the networks were fine-tuned by replacing the fully connected layer and the classification layer of these networks to fit the DME classification problem. The same setting of the input data was used to train each of the GoogLeNet, ResNet-50, Inception-v3, and SqueezeNet CNNs, separately. The networks were trained using the SGM algorithm with a maximum of 400 epochs, a mini-batch size of 4, and an initial learning rate of $4\times 10^{-4}$. The performance metrics of the aforementioned pretrained networks are reported in Table 2. The highest metrics of the proposed models are recorded in bold font and those of the original pretrained CNNs are underlined in Table 2. It is clear that the Inception-v3 network records the highest classification performance over the other three CNNs in terms of all measures. It achieves an accuracy of 94%, sensitivity of 97.1%, and specificity of 91.1%. SqueezeNet achieves the same sensitivity as the Inception-v3 network but reports a poor performance in the other two metrics. ResNet-50 is the worst performer over all the networks. By comparing the proposed system to these CNNs, it is obvious that the introduced system outperforms the Inception-v3 network and consequently the other three CNNs for model M1. We noticed that model M2 achieves a higher specificity and accuracy than Inception-v3 and SqueezeNet but a lower sensitivity. However, this model records a higher performance than the ResNet-50 and GoogLeNet networks in all metrics. Model M3 performs better than ResNet-50 in all metrics, and only in specificity and accuracy is it better than GoogLeNet, SqueezeNet, and Inception-v3. However, it records a sensitivity as high as GoogLeNet. Generally, the proposed feature transfer learning-based system outperforms the transfer learning classification using the original pretrained CNNs.

The performance of the proposed system was further evaluated against a number of state-of-the-art DME diagnosis systems. Model M1 was considered the representative of the presented DME systems in the comparison with other systems. The introduced system was compared to the systems developed by Abbas [17], Singh et al. [23], and Sulaiman et al. [25] because they used the same IDRiD dataset in training and testing their systems as in the present study. The system developed by Sulaiman et al. [25], in particular, was chosen for comparison because it was close to the proposed system. Both systems similarly preprocess the input dataset and use feature transfer learning and an autoencoder network for DME diagnosis. Nonetheless, the arrangement of the system developed by Sulaiman [25] opposes the presented system, and the feature extraction process is different as well. The work of Sulaiman et al. [25] employed a convolutional autoencoder to extract features from the input dataset cascaded by a DME-grading network. The autoencoder is pretrained using the Kaggle dataset of fundus images, and the grading network is composed of the encoders of the autoencoder connected to fully connected layers.

Table 3 compares the proposed DME system with the aforementioned systems. The comparison reveals that the introduced system achieves the highest accuracy and sensitivity over all three systems. Regarding the specificity, the introduced diagnosis system outperforms the systems in [17,25]; however, it performs almost the same as the system presented in [23]. In general, the proposed feature transfer-based autoencoder network system performs better than the three DME diagnosis systems.

## 5. Conclusions

In this paper, a new deep feature transfer learning-based stacked autoencoder system was proposed for the diagnosis of DME in color fundus images. The system utilizes deep feature extraction from an input dataset through four pretrained CNNs which are GoogLeNet, ResNet-50, SqueezeNet, and Inception-v3. The individual feature sets from all networks are combined together and used for classification. This strategy enables the generation of an enlarged comprehensive feature set from a limited size image input dataset. This grand feature set is then refined using the feature selection capability of autoencoder networks. The refined feature set, which contains the most important distinguishing features, is used to train a stacked autoencoder classifier in a semi-supervised manner to perform the diagnosis of DME. Three experiments were conducted to investigate the impact of the size of the refined feature set on the classification performance of the system. The results show that the enlargement of the training feature set improves the classification performance. The proposed deep feature transfer-based system achieves a maximum sensitivity of 97.5%, specificity of 95.5%, and accuracy of 96.8% using the IDRiD dataset. Moreover, the experimental results indicate that the proposed DFTSA-Net system outperforms the four original pretrained CNNs and state-of-the-art DME diagnosis systems.

## Figures and Tables

**Table 1:** Characteristics of the used pretrained CNNs.

CNN Name	Depth (Number of Layers)	Image Input Size	Number of Extracted Features/Image
GoogLeNet	22	224 × 224 × 3	1024
Inception-v3	48	299 × 299 × 3	2048
ResNet-50	50	224 × 224 × 3	2048
SqueezeNet	18	227 × 227 × 3	1000

**Table 2:** Classification performance metrics recorded by the proposed system and original pretrained CNNs; entries with bold font are for the best performance classifier; underlined entries are for the pretrained CNN achieving the highest performance only over the other pretrained CNNs.

Model	Size of RF1	Number of Features in RF1	Number of Features in RF2	Specificity %	Sensitivity %	Accuracy %
Proposed DFTSA-Net DME diagnosis system						
**M1**	**¾ GFS**	**4590**	**2295**	**95.5**	**97.5**	**96.8**
M2	½ GFS	3105	1553	95.4	96.8	96.2
M3	¼ GFS	1553	777	95.0	95.5	95.3
Pretrained CNNs						
-	GoogLeNet	-	-	85.8	95.5	90.3
-	SqueezeNet	-	-	73.1	97.1	85.1
-	Inception-v3	-	-	91.1	97.1	94.0
-	ResNet-50	-	-	62.7	92.5	77.6

**Table 3:** Comparison results of the proposed DFTSA-Net with state-of the-art DME diagnosis systems.

Method	Dataset	SP %	SN %	AC %	Publication
Feature pretraining dense convolutional neural network to diagnose DME without data preprocessing on gray images.	HEI-MED, IDRiD, and MESSIDOR	94.4	87.5	91.2	Abbas [17]
Hierarchical ensemble CNN with morphological preprocessing step on color fundus images.	IDRiD and MESSIDOR	95.8	96.3	96.1	Singh et al. [23]
Deep learning-based DME-grading model composed of a pretrained autoencoder followed by DME-grading network with data augmentation and preprocessing of color fundus images.	IDRiD	Not reported	68	68	Sulaiman et al. [25]
Deep feature transfer learning-based stacked autoencoder neural network for DME diagnosis with data augmentation and preprocessing of color fundus images.	IDRiD	95.5	97.5	96.8	The proposed system

## Data Availability

Data sharing is not applicable to this article as the authors used a publicly available dataset, whose details are included in the Section 3 of this article. Please contact the authors for further requests.

## Abbreviations

AC	Classification accuracy
CAD	Computer-aided diagnosis
CLAHE	Contrast-limited adaptive histogram equalization
CNN	Convolutional neural network
DCNN	Dense convolutional neural network
DFTSA-Net	Deep feature transfer-based stacked autoencoder network
DME	Diabetic macular edema
DR	Diabetic retinopathy
FCM	Fuzzy C-means
GFS	Grand feature set
HE-CNN	Hierarchical ensemble CNN
IDRiD	Indian Diabetic Retinopathy Image Dataset
IRC	Intraretinal cystoid fluid
OCT	Optical coherence tomography
PCA	Principal component analysis
SCGA	Scaled conjugate gradient algorithm
SGM	Stochastic gradient descent with momentum
SN	Sensitivity
SP	Specificity
SVM	Support vector machine
WHO	World Health Organization
